# Evaluating ChatGPT's Role in Patient Education on Distraction Osteogenesis for Limb Lengthening

**DOI:** 10.7759/cureus.114235

**Published:** 2026-08-09

**Authors:** Emma Eng, Elisheva Knopf, Nathan Gilmore, Anna Redden, Austin W Hansen, Samantha Reiss, Payton Yerke, Lisa Martinez

**Affiliations:** 1 Charles E. Schmidt College of Medicine, Florida Atlantic University, Boca Raton, USA; 2 College of Medicine, Florida Atlantic University, Boca Raton, USA; 3 Medicine, Charles E. Schmidt College of Medicine, Florida Atlantic University, Boca Raton, USA; 4 Orthopedic Surgery, Charles E. Schmidt College of Medicine, Florida Atlantic University, Boca Raton, USA; 5 Biomedical Sciences, Nova Southeastern University Dr. Kiran C. Patel College of Allopathic Medicine, Fort Lauderdale, USA; 6 Orthopedics, University of Central Florida, Orlando, USA; 7 Orthopaedics, Louisiana State University Health Sciences Center, Shreveport, USA

**Keywords:** chatgpt in healthcare, limb lengthening procedures, patient education, post-op pain management, post-procedure

## Abstract

Background: Limb lengthening requires patients to understand a complex procedure, a prolonged recovery, and potential complications. This study evaluated the quality, reliability, readability, and clinical completeness of ChatGPT-3.5 (OpenAI, San Francisco, CA) responses to common patient questions about limb lengthening.

Methodology: In this descriptive, cross-sectional content analysis, a purposive sample of 10 commonly encountered and clinically relevant questions was identified from the frequently asked questions pages of 10 healthcare institutions. Each question was entered separately into ChatGPT-3.5 on January 18, 2024, without follow-up prompts. Two senior authors independently evaluated each response against published literature and scored it with the Quality Criteria for Consumer Health Information (DISCERN) instrument, Journal of the American Medical Association (JAMA) benchmark criteria, and Flesch-Kincaid grade level. Outcomes were summarized descriptively, and interrater agreement was assessed with the Cohen kappa coefficient.

Results: The mean DISCERN score was 35.5 (range: 21-42). One response was rated very poor, seven were rated poor, and two were rated fair. Every response received a JAMA benchmark score of 0. The mean Flesch-Kincaid grade level was 13.6 (range: 12.3-15.7), indicating postsecondary reading demand.

Conclusions: ChatGPT-3.5 provided broad introductory information, but most responses lacked transparent sources, required clinical clarification, and were too complex for general patient education. It may be useful as a clinician-reviewed adjunct but should not replace individualized counseling by a qualified healthcare professional.

## Introduction

Limb lengthening uses distraction osteogenesis to increase bone length for congenital deformity, skeletal dysplasia, posttraumatic limb-length discrepancy, bone loss, and selected reconstructive indications [1]. Although the procedure has been performed for more than a century, techniques and implant options have evolved substantially. Patients considering treatment must understand the operation, the prolonged recovery process, and the possibility of complications.

Artificial intelligence (AI) tools are increasingly used to answer health questions. ChatGPT is a conversational large language model that can generate explanations without requiring users to navigate medical literature directly [2]. Patients considering limb lengthening may therefore use it to explore procedural and recovery questions. However, the quality, source transparency, readability, and clinical completeness of its answers in this subspecialty have not been established. This study evaluated a fixed set of 10 ChatGPT-3.5 responses to common patient questions about limb lengthening by using standardized quality and readability measures and comparison with published orthopedic literature.

## Materials and methods

Study design, setting, and sampling

This descriptive, cross-sectional content-analysis study evaluated a fixed sample of ChatGPT-3.5 responses. A purposive, non-probability sampling technique was used to identify 10 commonly encountered and clinically relevant patient questions from the frequently asked questions sections of 10 healthcare institution webpages. The webpages were selected purposively because they contained publicly accessible limb-lengthening FAQ content; a systematic search protocol, reproducible search string, formal institution-level eligibility framework, and screening log were not documented. The sampling frame was the FAQ content available on those selected webpages. Questions were eligible when they addressed clinical information directly relevant to a patient considering limb lengthening, including achievable length, the procedure, recovery, device use and adjustment, complications, indications, return to activity, or growth arrest. Administrative, billing, and scheduling questions were excluded. Question selection relied on investigator judgment; a systematic patient survey, validated question-selection instrument, and formal consensus process were not used. No participant-level inclusion or exclusion criteria applied because no people or records were enrolled. All data were generated and evaluated online, so there was no clinical study site or geographic patient population.

Data collection

ChatGPT data collection occurred on January 18, 2024, making this a single-day snapshot of one model version. Each question was entered separately and once into ChatGPT-3.5 (OpenAI, San Francisco, CA) through its free web interface. The question alone served as the prompt. No follow-up questions, requests for citations, or prompts for clarification were used, and each response was recorded for analysis. The exact wording of all 10 prompts is reproduced in Table 1.

**Table 1:** Quality and readability scores for the 10 frequently asked questions. DISCERN scores range from 16 to 80; higher scores indicate better-quality written treatment information. Ratings used in this study were very poor (16-29), poor (30-40), fair (41-51), good (52-63), and excellent (64-80). JAMA benchmark scores range from 0 to 4; one point is awarded for authorship, attribution, disclosure, and currency. Flesch-Kincaid grade level estimates the U.S. school grade level required to understand the text. JAMA, Journal of the American Medical Association

Question	DISCERN score (16-80)	DISCERN rating	JAMA benchmark score (0-4)	Flesch-Kincaid grade level
1. How much height am I able to gain with surgical limb lengthening?	36	Poor	0	12.3
2. What is the recovery process after surgical limb lengthening?	31	Poor	0	14.8
3. How do you surgically lengthen a bone?	42	Fair	0	13.4
4. How do the intramedullary nail and external fixator work in limb lengthening?	42	Fair	0	12.9
5. What does the surgery for limb lengthening entail?	39	Poor	0	12.4
6. How are the adjustments to the limb lengthening devices performed?	39	Poor	0	13.1
7. What are the potential complications of surgery for limb lengthening?	38	Poor	0	14.0
8. What conditions are treated by limb lengthening?	32	Poor	0	13.7
9. When can I return to normal activities after surgical limb lengthening?	35	Poor	0	14.1
10. When is the growth plate arrested in surgical limb lengthening?	21	Very poor	0	15.7

Outcome measures and statistical analysis

The response was the unit of analysis. Two senior authors independently compared each response with current published literature and, consistent with an earlier orthopedic ChatGPT study [3], evaluated quality and reliability with DISCERN [4], source transparency with the Journal of the American Medical Association (JAMA) benchmark criteria [5], and readability with the Flesch-Kincaid grade level [6]. DISCERN totals were assigned qualitative ratings ranging from very poor to excellent; the JAMA benchmark was scored from 0 to 4, and the Flesch-Kincaid result was reported as a U.S. school grade level. Only aggregate scores and ratings are reported; the wording of instrument items is not reproduced.

The clinical comparison set comprised the peer-reviewed orthopedic publications cited in the question-specific analyses. It included topic-level and systematic reviews as well as primary cohort and comparative studies. No single clinical practice guideline or universal reference standard encompassed all 10 questions, and a formal evidence hierarchy was not prospectively specified.

Continuous results were summarized with the mean, median, range, and interquartile range (IQR); IQRs were calculated from the 25th and 75th percentiles using linear interpolation. Qualitative rating categories were summarized with counts. Interrater agreement was assessed with the Cohen kappa coefficient. No hypothesis tests, p-values, group comparisons, regression models, or other inferential statistical tests were used.

No a priori sample size calculation or power analysis was performed because this was an exploratory descriptive analysis of a fixed 10-question sample rather than a hypothesis-testing study. Conventional confounding was not applicable because no exposure-outcome association or between-group effect was estimated. Potential sources of measurement variability were reduced by using the same model version, data-collection date, prompt-entry procedure, and no follow-up prompts for all questions; reviewer-related variability was evaluated through independent scoring and the Cohen kappa coefficient.

Ethical approval

This study analyzed publicly available AI-generated text and did not involve human participants or animals; institutional review board approval was therefore not required. No external funding was received.

## Results

The mean DISCERN score was 35.5 (range: 21-42), with a median of 37.0 (interquartile range (IQR): 32.8-39.0). One response was rated very poor, seven were rated poor, and two were rated fair. Every response received a JAMA benchmark score of zero (median: 0; IQR: 0-0). The mean Flesch-Kincaid grade level was 13.6 (range: 12.3-15.7), with a median of 13.6 (IQR: 13.0-14.1), indicating postsecondary reading demand. The Cohen kappa coefficient between the two raters was 0.783, indicating substantial agreement. Table 1 summarizes the scores for all 10 questions.

Across the responses, ChatGPT-3.5 generally described broad limb-lengthening concepts correctly, including variation in achievable length and the major phases of distraction osteogenesis. However, the responses often lacked sufficient qualification and individualized clinical context. Achievable length varies by indication, skeletal maturity, segment, and complication management [1,7-9], while the recovery-related responses underemphasized external-fixator pin-tract surveillance, individualized physical therapy, and the potential for persistent strength deficits [10,11].

Procedural and device explanations generally identified osteotomy, fixation, distraction, consolidation, external fixation, and intramedullary lengthening nails but incompletely addressed the biological basis of distraction osteogenesis, device selection, and device-specific burdens and complications [1,12-17]. Guidance regarding device adjustment appropriately emphasized scheduled monitoring, but greater emphasis was needed on avoiding independent changes to the prescribed distraction rate and modifying treatment according to radiographic healing, pain, joint motion, and neurovascular findings; shared decision-making is relevant when treatment options involve prolonged recovery and different risk profiles [13,17,18].

Indications were broadly described but required clearer distinction between common and highly selected reconstructive uses, with management varying by age, anatomy, predicted discrepancy, and patient goals [19-21]. Return-to-activity guidance appropriately avoided a universal timeline because healing and functional recovery vary by segment, protocol, and patient population [9,22-24]. The most clinically important deficiency involved the growth-plate question: the response did not clearly distinguish distraction osteogenesis from epiphysiodesis, whose timing depends on remaining growth and predicted discrepancy at skeletal maturity [21,25,26]. The wording of this prompt itself was ambiguous because it conflated epiphysiodesis with distraction osteogenesis; therefore, interpretation of this response should be cautious.

## Discussion

This analysis identified three recurring limitations in ChatGPT-3.5 responses about limb lengthening: poor source transparency, incomplete clinical context, and advanced readability. The chatbot generally introduced major concepts correctly, but several answers required qualification to avoid overly broad or misleading conclusions.

The absence of verifiable authorship, attribution, disclosure, and currency information explains the zero JAMA benchmark score for every response and also lowered DISCERN ratings. These measures characterize the quality, reliability, and source transparency of patient-facing information but do not independently establish the clinical accuracy of every statement [3]. For that reason, the qualitative comparison with orthopedic literature was necessary to identify clinically important omissions and overgeneralizations.

The readability findings also limit independent patient use. The responses required postsecondary reading ability, which is above commonly recommended targets for patient education. Orthopedic studies have similarly found that unmodified ChatGPT output often remains too complex, may omit relevant context, and should be reviewed before use in patient counseling [3,27,28]. Previously published research, rather than the present study, suggests that targeted prompting can improve readability; however, simplification still requires professional review to preserve clinical completeness [29].

These findings support a limited adjunctive role for conversational AI in limb-lengthening education. A clinician-reviewed response may help introduce terminology and prepare patients for discussion, but the model should not be treated as an authoritative or individualized source. Based on the authors' interpretation of these findings, future patient-facing systems should identify sources, disclose uncertainty, and consistently direct patients to qualified professionals when recommendations depend on age, anatomy, diagnosis, implant choice, or complication risk; these recommendations were not directly tested as outcomes in the present study.

Limitations

This study evaluated only 10 questions, one model version, one response generation per question, and one point in time. Large language model responses can change after software updates and may vary across sessions, so these findings may not generalize to later versions, repeated generations, or differently worded prompts.

The question set was derived from institutional frequently asked questions pages rather than a systematic survey of patients, and selection relied on investigator judgment rather than a validated question-selection instrument or formal consensus process. This may introduce selection bias and may not capture every clinically important patient concern. The prompts were intentionally entered without follow-up requests, whereas patients may refine a question during real-world use.

Although standardized instruments were used, qualitative comparison with published literature required clinical judgment. Two senior authors scored the responses independently, but some subjectivity remains. In addition, Flesch-Kincaid grade level estimates linguistic complexity and does not directly measure comprehension, accuracy, or usefulness. The study also did not directly evaluate patient understanding, perceived usefulness, satisfaction, or effects on decision-making.

## Conclusions

ChatGPT-3.5 can provide a broad introduction to limb-lengthening procedures, but its source transparency, readability, and clinical completeness were insufficient for independent patient education. It may be used as a clinician-reviewed adjunct rather than a substitute for individualized counseling. Patient-facing AI content should identify sources, communicate uncertainty, and use accessible language. Further research should directly compare ChatGPT-3.5 with newer large language models, including GPT-4-class systems, using standardized prompts, multiple independent generations per prompt, broader and more representative question sets, and patient-centered outcomes such as understanding, usefulness, satisfaction, and decision-making.
